# Cytoreductive Surgery and Hyperthermic Intraperitoneal Chemotherapy with Intra-Operative Radiotherapy for Patients with Locally Advanced or Locally Recurrent Rectal Cancer and Peritoneal Metastases

**DOI:** 10.3390/cancers15030858

**Published:** 2023-01-30

**Authors:** Vincent C. J. van de Vlasakker, Teun B. M. van den Heuvel, Anouk Rijken, Simon W. Nienhuijs, Stijn H. J. Ketelaers, An-Sofie E. Verrijssen, Harm J. Rutten, Grard A. P. Nieuwenhuijzen, Jacobus W. A. Burger, Ignace H. J. T. de Hingh

**Affiliations:** 1 Department of Surgery, Catharina Hospital, 5623 EJ Eindhoven, The Netherlands; 2 Department of Radiotherapy, Catharina Hospital, 5623 EJ Eindhoven, The Netherlands; 3 GROW–School for Oncology and Reproduction, Maastricht University, 6211 LK Maastricht, The Netherlands; 4 Department of Research, Netherlands Comprehensive Cancer Organization, P.O. Box 19079, 3501 DB Utrecht, The Netherlands

**Keywords:** colorectal neoplasms, peritoneal metastases, cytoreduction, surgical procedures, CRS-HIPEC, intro operative radiotherapy, IORT, locally recurrent rectal cancer, LRRC, locally advanced rectal cancer, LARC

## Abstract

**Simple Summary:**

Although relatively rare, locally advanced or recurrent rectal cancer (LARC and LRRC) can metastasize. Patients whose advanced rectal cancer has metastasized to the peritoneum face a very poor prognosis. In selected patients, the prognosis might be improved by undergoing very intense treatment. This multimodality treatment, consisting of intraoperative radiotherapy (IORT), cytoreductive surgery, and hyperthermic intraperitoneal chemotherapy (CRS-HIPEC), is aimed at attaining a microscopic radical resection of the rectal tumor and its peritoneal metastases (PM), as this is the only option for long-term survival. The present study reports on 30 consecutive patients who have undergone this multimodality treatment. The results in terms of complications and survival are comparable to the results described in the literature on IORT and CRS-HIPEC as separate treatment entities. Thus, this multimodality treatment can be considered a treatment option for highly selected patients, provided that it is performed in a tertiary referral center.

**Abstract:**

*Purpose*: To assess the safety and long-term outcome of a multimodality treatment consisting of radical surgery, intra-operative radiotherapy (IORT), and cytoreductive surgery with hyperthermic intraperitoneal chemotherapy (CRS-HIPEC) for patients with locally advanced rectal cancer (LARC) or locally recurrent rectal carcinoma (LRRC) and peritoneal metastases (PM). *Methods*: The present study was a single-center cohort study, including all consecutive patients undergoing this treatment in a tertiary referral center for LARC, LRRC, and PM. Postoperative complications, intensive care stay (ICU stay), and re-admission rates were assessed as well as disease-free survival (DFS) and overall survival (OS). *Results*: A total of 14 LARC and 16 LRRC patients with PM were included in the study. The median ICU stay was 1 day, and 57% of patients developed a severe postoperative complication. No 90-day mortality was observed. Median DFS was 10.0 months (Interquartile Range 7.1–38.7), and median OS was 31.0 months (Interquartile Range 15.9–144.3). *Conclusions*: As postoperative complications and survival were in line with treatments that are accepted for LARC or LRRC and PM as separate procedures, we conclude that combined treatment with IORT and CRS-HIPEC should be considered as a treatment option for selected patients with LARC or LRRC and peritoneal metastases in tertiary referral centers.

## 1. Introduction

Colorectal cancer (CRC) is the third most common cancer worldwide, with a yearly incidence of 1.8 million cases [1,2]. Approximately one-third of CRCs are located in the rectum, of which up to 10% are diagnosed as locally advanced rectal cancer (LARC). LARC is defined as rectal cancer that invades or extends close to the mesorectal fascia or other circumjacent structures [3,4]. In addition to aggressive local growth, rectal cancer may also reoccur within the pelvis after previous surgical resection, giving rise to locally recurrent rectal cancer (LRRC) [5,6]. Both ARC and LRRC can metastasize systemically as well as to the peritoneal cavity. Peritoneal metastases (PM) occur in approximately 4% of patients with rectal cancer [7,8].

In patients with LARC or LRRC, multimodality treatment, including neo-adjuvant chemo- and/or radiotherapy, radical surgery, and intra-operative radiotherapy (IORT), is often required to accomplish a radical resection in order to attain adequate survival [4,9,10,11]. Selected patients with PM from colorectal cancer are treated by radical resection of PM by cytoreductive surgery (CRS) combined with hyperthermic intraperitoneal chemotherapy (HIPEC) [12,13,14,15]. The rationale for this treatment modality is the hypothesis that PM is a locoregional disease spreading rather than system dissemination. The selection of patients for this treatment is based on performance status, the extent of peritoneal disease, and the absence of systemic metastases [12,13,16,17,18].

As a consequence, to increase long-term survival, patients with LARC and PM or LRRC and PM-selected patients may benefit from a multimodal approach as well, including neoadjuvant chemoradiotherapy, extensive surgery, IORT, and CRS-HIPEC. However, as both IORT and CRS-HIPEC are only performed in specialized centers and the incidence of LRRC and PM and LRRC and PM is relatively low, this multimodality treatment is very rare.

Previously, we reported on the feasibility of this highly invasive, complication-prone, and demanding multimodal treatment regimen in five consecutive cases [19]. This treatment was concluded to be feasible, but the sample size was too small and the follow-up too short to meaningfully analyze safety and oncological outcomes.

The present study aims to assess the safety and oncological outcome of this multimodality procedure in a larger cohort of patients diagnosed with LARC or LRRC and PM.

## 2. Materials and Methods

The present study was a single-center cohort study performed in a Dutch tertiary referral center specializing in the treatment of both rectal cancer and PM. Patients were included in a prospectively maintained database between 1st January 2007 and 1st March 2022 and were included in the present study if both IORT and CRS-HIPEC were performed simultaneously in an elective setting or if CRS-HIPEC was performed within one week after surgery and IORT in an elective setting. IORT and CRS-HIPEC regimens were performed in accordance with local protocols in effect at the time, meaning only patients with WHO status 0–1 were deemed eligible, given the demanding nature of this multi-modality treatment. Partial CRS-HIPEC procedures (e.g., debulking, primary tumor resection, or application of HIPEC regimen only to the pelvis) were ground for exclusion. Depending on the extensiveness of the peritoneal disease, either peritonectomy or (multi-) visceral resections were performed as part of the cytoreductive surgery. The prospective database, and this study as an extension, were approved by a Central Ethics Committee (MEC-U, Nieuwegein, The Netherlands; Niet-WMO 2015-11) and conducted in accordance with the Declaration of Helsinki.

Baseline characteristics were derived from the characteristics of the primary tumor before treatment and were compared between patients with LARC and PM, and LRRC and PM. All patients who underwent multimodality treatment consisting of IORT and CRS-HIPEC were included in the safety analyses, which included the following parameters: duration of post-operative ICU stay, occurrence and severity of postoperative complications according to the Clavien Dindo classification (CD) [20], and re-admission within 90 days after the operative procedure. CD complications were scored until 90 days after surgery, and only the highest CD grade complication that occurred was reported for each patient, and complications were regarded as severe if they were CD grade 3 or higher. Safety parameters were compared between patients with LARC and PM and patients with LRRC and PM. Differences in continuous variables between the two groups were compared using Mann-Whitney U tests and presented as median (interquartile range). Differences in categorical variables between the two tumor-onset groups were compared using χ2 tests and presented as n (%). Missing data were not included in the comparative analyses.

For the survival analyses, patients with isolated peritoneal metastases were included in whom a complete macroscopic cytoreduction was attained (CC0) with a minimum follow-up of 3 months. The Kaplan-Meier method was used to estimate the interval from CRS-HIPEC to disease recurrence for disease-free survival (DFS) or death for overall survival (OS). Log-rank tests were performed to assess differences in DFS and OS between patients with LARC and PM and patients with LRRC and PM.

All tests were performed in a two-sided fashion, and *p* < 0.05 was considered statistically significant. All analyses were performed using SPSS Statistics for Windows, Version 25.0. Armonk, NY, USA: IBM Corp.

## 3. Results

During the study period, a total of 30 patients with LARC and PM (n = 14) or LRRC and PM (n = 16) were treated with IORT and CRS-HIPEC and were thus included in the present study. All patients were included in the safety analyses, whereas 25 patients were included in the survival analyses. Reasons for exclusion from the survival analyses were a follow-up period of fewer than three months after the surgical procedure (i.e., patients treated after January 2022 and were thus too close to the date of final data collection) (n = 2), macroscopic incomplete cytoreduction (n = 1), and the presence of at least one systemic metastasis at the time of CRS-HIPEC treatment (n = 2).

Baseline characteristics are presented in Table 1. The majority of patients were male (63%), had an ASA score of 2 (77%), and did not smoke (73%). The mean age was 57.9 years. The extent of peritoneal disease load was assessed through the peritoneal cancer index (PCI) as first described by Sugarbaker [17], and the mean PCI score was 7.4. PCI score was the only baseline characteristic that significantly differed between the LARC and LRRC groups (5.4 vs. 9.1, respectively; *p* = 0.016).

### 3.1. Treatment

The different treatment strategies are presented in Table 2. All patients received neoadjuvant treatment, most commonly chemoradiotherapy (80%). The mean operation time was 437 min, and the mean amount of blood loss was 2062 mL. A majority of patients received mitomycin-C-based HIPEC treatment (n = 28), with only two patients who were treated with oxaliplatin-based HIPEC. Both HIPEC regimens had a standard protocol of treatment: mitomycin-C was administered for 90 min and heated to 42 degrees Celsius, and oxaliplatin for 30 min at 42 degrees Celsius. Most patients received an IORT-dosage of 10 Gray at the area most at risk for irradicality (90%). Treatment characteristics did not differ significantly between both groups. However, the amount of blood loss seemed more in the LRRC group when compared to the LARC group (2552 mL vs. 1501 mL, respectively; *p* = 0.057).

### 3.2. Safety

The outcomes of the safety analyses are presented in Table 3. The median postoperative length of intensive care unit (ICU) stay was one day (IQR 1–3 days). None of the patients with LARC and PM were re-admitted to the ICU for postoperative complications, compared to 31% of patients with LRRC and PM. Severe postoperative complications occurred in 57% of all patients. Severe complications occurred significantly more frequently in patients in the LRRC and PM groups (75% vs. 36%, respectively, *p* = 0.01). Intra-abdominal abscesses requiring percutaneous drainage were the most commonly observed postoperative complications (occurring in 33% of patients). Postoperative mortality within 90 days did not occur. The re-admission rate within 90 days was 27% for all patients and similar between both groups (29% in the LARC and PM group vs. 25% in the LRRC and PM group).

### 3.3. Survival

**Figure 1:**
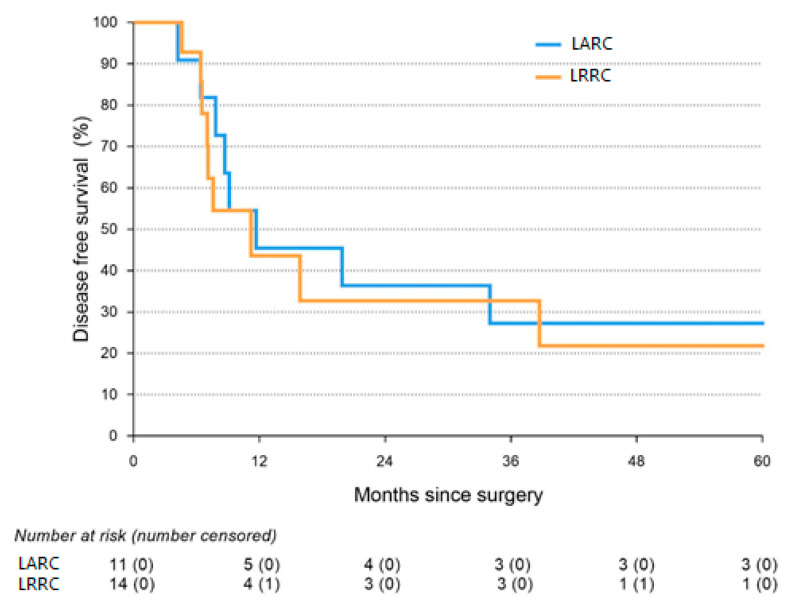
Kaplan-Meier graph of disease-free survival.

**Figure 2:**
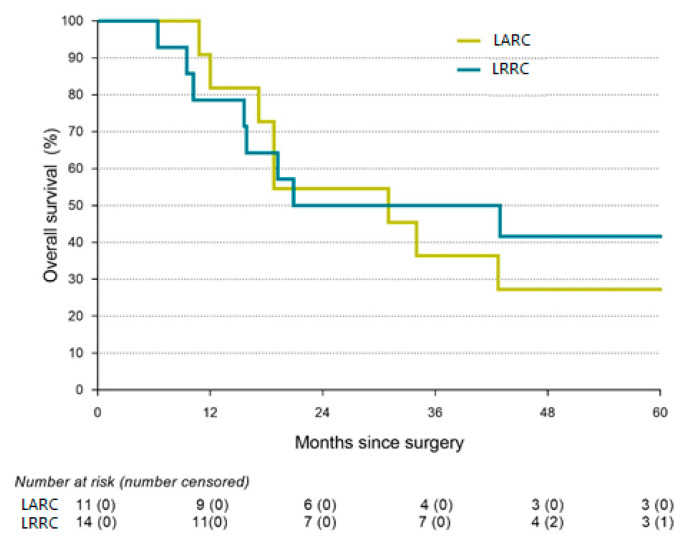
Kaplan-Meier graph of overall survival.

Survival analyses are presented in Table 4. Kaplan-Meier curves of the OS and DFS are presented in Figure 1 and Figure 2. The Median OS of the total study population was 31.0 months (IQR: 6.4–not reached), while the median DFS of the total study population was 10.0 months (IQR: 15.9–144.3). Neither OS (31.0 months vs. 20.9 months, respectively; log-rank, *p* = 0.382) nor DFS (11.7 months vs. 10.0 months, respectively; log-rank, *p* = 0.123) differed significantly between both groups.

Furthermore, three patients in the LARC and PM group and one patient from the LRRC and PM group were alive and without disease recurrence five years after finishing treatment and were discharged from further follow-up.

## 4. Discussion

In the present study, 30 consecutive patients presented who were diagnosed with locally advanced rectal cancer and peritoneal metastases or with locally recurrent rectal cancer and peritoneal metastases. All patients received an intensive treatment approach consisting of neo-adjuvant chemo-radiotherapy, radical surgery, IORT, and CRS-HIPEC. It was observed that severe postoperative complications were common in both groups, but that postoperative mortality did not occur in either group. The median OS was 31 months for the group as a whole and did not differ significantly between the LARC and the LRRC group.

Microscopic radical (R0) resection is the main goal in the treatment of LARC and LRRC, as it offers the best prognosis in terms of long term-survival and sometimes even offers a cure [21]. In LARC and LRRC, surgery alone is usually not enough to attain a microscopic radical resection. Therefore, neo-adjuvant strategies, including radiotherapy and/or chemotherapy, have been introduced to downstage the tumor, observe the biological behavior and facilitate a radical resection. IORT, as an additional treatment, consists of a single boost of radiation therapy that is delivered during surgery to the area most at risk for microscopic residual disease. This enables the delivery of a higher dose of radiation to at-risk areas while sparing healthy structures. IORT in our center was introduced in 1994 and is delivered using electron beams. Until 2016 this was performed using an Elektra SL-25 linear accelerator (Elektra Oncology Systems, Stockholm, Sweden) and from 2016 onward, using a Mobetron 2000 linear accelerator (IntraOp Inc, Sunnyvale, CA, USA). The bevel angle (0-, 30- or 45-degrees), the diameter of the tube (5–7 cm), and energy used (6–12 MeV) depend on the target area, tissue at risk, and body anatomy, while the prescribed dose generally ranges from 10 Gy to 12.5 Gy depending on the expected extent of residual dose. As of September 2021, an adapted IORT technique was applied in our institute in order to deliver a higher surface dose to the at-risk area. From this moment on, only a prescribed dose of 10 Gy was delivered, providing a surface dose of 15.5–17.0 Gy depending on the energy, bevel, and size of the tube used.

Over the years, this IORT regimen has resulted in a median OS of 41 months for patients with LARC and 31 months for patients with LRRC [22]. Complication rates of severe postoperative complications in our center are 30% in LARC patients and 46% in LRRC patients [22]. This increased complication rate in LRRC patients may be a result of the higher PCI score and its corresponding higher blood loss and extensiveness of the procedure. Furthermore, it is known that previous surgery often results in more adhesions and, subsequently, in a more challenging procedure [4,22]. This is supported by the results in Table 2 (Treatment characteristics). Both OS and complication rates are slightly better in the literature than those described in the present study. 

Although relatively rare, rectal cancer may metastasize to the peritoneal cavity, giving rise to peritoneal metastases [7]. In both colon- and rectal cancer, the presence of these peritoneal metastases has a profound negative impact on survival. In selected cases, radical resection of these metastases, a procedure commonly referred to as cytoreductive surgery, has been shown to improve survival. The addition of short-course oxaliplatin-based HIPEC has been under debate since the PRODIGE7 trial, but Mitomycin-C based HIPEC, which was then applied regimen in the majority of patients in the present study, remains the standard of care in The Netherlands and various countries around the world [23,24].

CRS-HIPEC was shown to improve survival in selected patients with the colorectal PM as compared with palliative (5-fluorouracil-based) systemic therapy and palliative surgery in an RCT published in 2003 [25,26]. Since then, CRS-HIPEC has resulted in an OS in The Netherlands of almost three years [27]. CRS-HIPEC was introduced in our center in 2007 and has since resulted in an OS that is comparable to the Dutch OS, with a median OS of 35 months for patients with colorectal peritoneal metastases (CPM) treated through CRS-HIPEC [13]. 

For non-advanced rectal cancer patients with PM specifically, treatment through CRS-HIPEC in our center has resulted in a median OS of 26 months, with a corresponding median DFS of 13.5 months [28]. Interestingly, OS described in the present study is longer, while no significant difference was observed between LARC and PM, and LRRC and PM patients. This indicates that PM is the limiting factor for OS rather than the status of the rectal tumor. Moreover, it indicates that the multimodality treatment described in the present study can be regarded as effective.

Furthermore, approximately 30% of patients with non-advanced rectal cancer and PM who undergo CRS-HIPEC develop severe postoperative complications [28]. This complication rate is slightly better than the presented complication rate of the current study. 

Given the intensive character of the multimodality treatment, postoperative complications are to be expected. Only two case series reporting on patients undergoing both IORT and CRS-HIPEC have been published previously, one on patients with pancreatic cancer and one on five colorectal cancer patients, who are all part of the present study’s population [19,29]. Therefore, comparing our results to those of other studies that investigated the treatment of, treating similar patients is not possible. Nevertheless, both the number of complications and survival in the current cohort are in line with studies that investigated CRS-HIPEC for the treatment of PM and IORT as a treatment for LARC and LRRC as separate treatment modalities. The higher complication rate in patients with LRRC and PM that was observed in the present study may be explained by the fact that these patients had already undergone previous abdominal surgery and had a higher PCI score, thereby rendering the necessary CRS more difficult due to adhesions and more extensive due to the extent of the peritoneal disease-load.

The specific role and effect of IORT as a treatment in rectal cancer are still under investigation [30]. Nonetheless, currently, both radical resection and IORT for LARC and CRS and HIPEC for PM have accepted multimodality treatments considered to be safe and effective when performed in high-volume expert centers. As the complications and survival outcomes for both patients with LARC and PM as well as patients with LRRC and PM in the current study are in line with the literature on these treatments separately, we conclude that the multimodality treatment as presented in this study should also be regarded as safe and effective, especially considering disease severity and treatment intensity.

Evidently, there are some limitations to this study. First, patients were highly selected as this treatment is highly invasive and demanding. This is reflected in the relatively young age of the included patients and the relatively low PCI score. As a result, few patients are selected, leading to a long inclusion period, inducing a potential bias, as the preoperative process (e.g., diagnostics, treatments, and staging) in rectal cancer has greatly evolved in the last decades. Moreover, this multi-modality treatment approach requires a highly trained and specialized medical team as well as specialized equipment, rendering this multimodality treatment not readily available for affected patients. Finally, although complication rates are acceptable, there is no information on the quality of life and functional outcomes of these patients.

## 5. Conclusions

In conclusion, the combination of neo-adjuvant therapy, extensive surgery, IORT, CRS, and HIPEC for patients with LARC and PM or LRRC and CPM is feasible and safe and results in relatively long term-survival and even cure in highly selected patients. Although this invasive multimodality treatment is accompanied by significant morbidity, it can still be considered a viable treatment option for selected patients, provided it is performed in a specialized treatment center. Further research to optimize patient selection and treatment is warranted.

## Figures and Tables

**Table 1:** Baseline characteristics of the study population.

	Total Group (n = 30)	LARC (n = 14)	LRRC (n = 16)	*p*-Value
**Sex**				
Male	19 (63)	9 (64)	10 (62.5)	0.919
Female	11 (37)	5 (36)	6 (37.5)	
**Age**				
(in years)	57.9 ± 11	56.8 ± 10.8	58.8 ± 11.5	0.547
**ASA-score**				
1	2 (7)	2 (14)	0 (0)	0.291
2	23 (77)	10 (71)	13 (81)	
3	5 (17)	2 (14)	3 (19)	
**Smoking**				
No	22 (73)	9 (64)	13 (81)	0.295
Yes	8 (27)	5 (36)	3 (19)	
**T-stage**				
2	1 (3)	0 (0)	1 (6)	0.158
3	15 (50)	5 (36)	10 (63)	
4	14 (47)	9 (64)	5 (31)	
**N-stage**				
0	9 (30)	3 (21)	6 (38)	0.141
1	13 (43)	5 (36)	8 (50)	
2	8 (27)	6 (43)	2 (13)	
**PCI-score**	7.4 ± 5.0	5.4 ± 5.0	9.1 ± 4.5	0.016

All values are mean ± standard deviation, or n (%); ASA American society of anesthesiologists score; T tumor stage; N nodal stage; PCI peritoneal cancer index score; LARC locally advanced rectum cancer; LRRC locally recurrent rectal cancer.

**Table 2:** Treatment characteristics.

	Total Group (n = 30)	LARC (n = 14)	LRCC (n = 16)	*p*-Value
**Neoadjuvant treatment**				
Radiotherapy	2 (7)	0 (0)	2 (13)	0.218
Chemotherapy	4 (13)	1 (7)	3 (19)	
Chemoradiotherapy	24 (80)	13 (93)	11 (69)	
**Duration of the operative procedure**				
(in minutes)	438 ± 75	427 ± 83	447 ± 68	1.000
**Blood loss**				
(in mL)	2062 ± 1328	1501 ± 937	2552 ± 1449	0.057
**IORT-dose**				
10 Gray	27 (90)	13 (93)	14 (87.5)	0.626
12.5 Gray	3 (10)	1 (7)	2 (12.5)	
**HIPEC-regime**				
Mitomycin C	28 (93)	14 (100)	14 (87.5)	0.171
Oxaliplatin	2 (7)	0 (0)	2 (12.5)	

All values are mean ± standard deviation, or n (%); IORT intraoperative radiotherapy; HIPEC Hyperthermic intraperitoneal chemotherapy; LARC locally advanced rectum cancer; LRRC locally recurrent rectal cancer.

**Table 3:** Outcome measures of safety.

	Total Group (n = 30)	LARC (n = 14)	LRRC (n = 16)	*p*-Value
**Postoperative ICU stay** (in days)	1 (1–3)	1 (1–2)	1 (1–3)	0.423
**Highest Severity of complications, according to the Clavien-Dindo classification**				
CD 0–2	13 (43)	9 (64)	4 (25)	0.010
CD 3	12 (40)	5 (36)	7 (44)	
CD 4	5 (17)	0 (0)	5 (31)	
CD 5	0 (0)	0 (0)	0 (0)	
**Readmission within 90 days**				
No	22 (73)	10 (71)	12 (75)	0.825
Yes	8 (27)	4 (29)	4 (25)	

All values are median (interquartile range), or n (%); ICU Intensive care unit; CD Clavien-Dindo; LARC locally advanced rectum cancer; LRRC locally recurrent rectal cancer.

**Table 4:** Outcome measures of survival.

	Total Group (n = 25)	LARC (n = 11)	LRRC (n = 14)	*p*-Value
**Disease-free survival**				
in months	10.0 (6.4–38.7)	11.7 (7.8– *)	10.0 (6.4–38.7)	0.123
Site of recurrence **				0.723
No recurrence	8	4	4	
Local	1	0	1	
Locoregional	10	5	5	
Systemic	6	2	4	
**Overall survival**				
in months	31.0 (15.9–144.3)	31.0 (17.2–144.3)	20.9 (15.6–81.3)	0.123

All values are median (interquartile range; LARC locally advanced rectum cancer; LRRC locally recurrent rectal cancer; * Endpoint not reached. ** Local is defined as the site of IORT, locoregional as the peritoneal cavity, and systemic as other sites of disseminated disease.

## Data Availability

Data will be readily made available upon written request to the corresponding author.
